# National Trends in the Use of Neoadjuvant Therapy Before Cancer Surgery in the US From 2004 to 2016

**DOI:** 10.1001/jamanetworkopen.2021.1031

**Published:** 2021-03-10

**Authors:** Christopher T. Aquina, Aslam Ejaz, Allan Tsung, Timothy M. Pawlik, Jordan M. Cloyd

**Affiliations:** 1 Division of Surgical Oncology, Department of Surgery, The Ohio State University Wexner Medical Center, Columbus

## Abstract

This cohort study uses information obtained from the US National Cancer Database to evaluate trends in the use of neoadjuvant therapy before tumor resection for various types of cancer from 2004 to 2016.

## Introduction

A major contributor to improved oncologic survival is an emphasis on multimodal therapies. Although surgery followed by adjuvant therapy has traditionally been considered the standard approach to most solid-organ cancers, administration of neoadjuvant therapy (NT) has several benefits. Neoadjuvant therapy allows for early treatment of micrometastatic disease, improved patient selection with favorable tumor biologic features for surgery, and tumor downstaging and is associated with improved completion rates of multimodal therapy compared with adjuvant therapy.^1,2,3,4^ However, whether these potential benefits are associated with an increase in the adoption of NT remains unknown. In this cohort study, the National Cancer Database (NCDB) was used to identify national trends in use of NT for cancer diagnoses from January 1, 2004, to December 31, 2016.

## Methods

Records for patients diagnosed with primary breast, esophageal, gastric, pancreatic, rectal, extremity sarcoma, bladder, or ovarian cancer between January 1, 2004, and December 31, 2016, who underwent curative-intent resection were identified in the NCDB. The NCDB is an oncology database that contains patient-level information for more than 1500 hospitals accredited by the Commission on Cancer in the US and represents 70% or more of newly diagnosed cancer cases nationwide.^5^ Patients with distant metastases or missing treatment information were excluded from all analyses, and patients with other missing data were excluded from multivariable analyses. Institutional review board approval and the requirement for informed consent were waived by The Ohio State University institutional review board because data were deidentified. This study followed the Strengthening the Reporting of Observational Studies in Epidemiology (STROBE) reporting guideline.

Trends in the use of NT, including chemotherapy, radiotherapy, and hormone therapy, were analyzed. The Cochran-Armitage test for trend and mixed-effects logistic regression, which included the unique hospital identifier as a clustering variable, were performed using SAS, version 9.4 (SAS Institute) and R, version 3.6.3 (R Foundation for Statistical Computing), respectively. Significance testing was 2-sided, and *P* < .05 was considered to be statistically significant.

## Results

**Figure 1.**
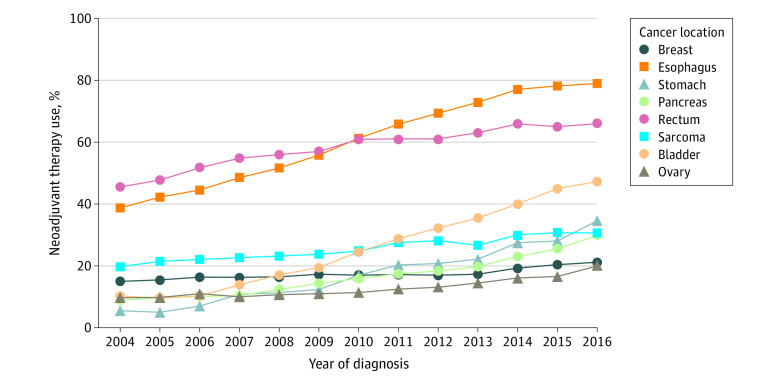
Trends in the Use of Neoadjuvant Therapy for Various Cancer Types in the US From 2004 to 2016

**Figure 2.**
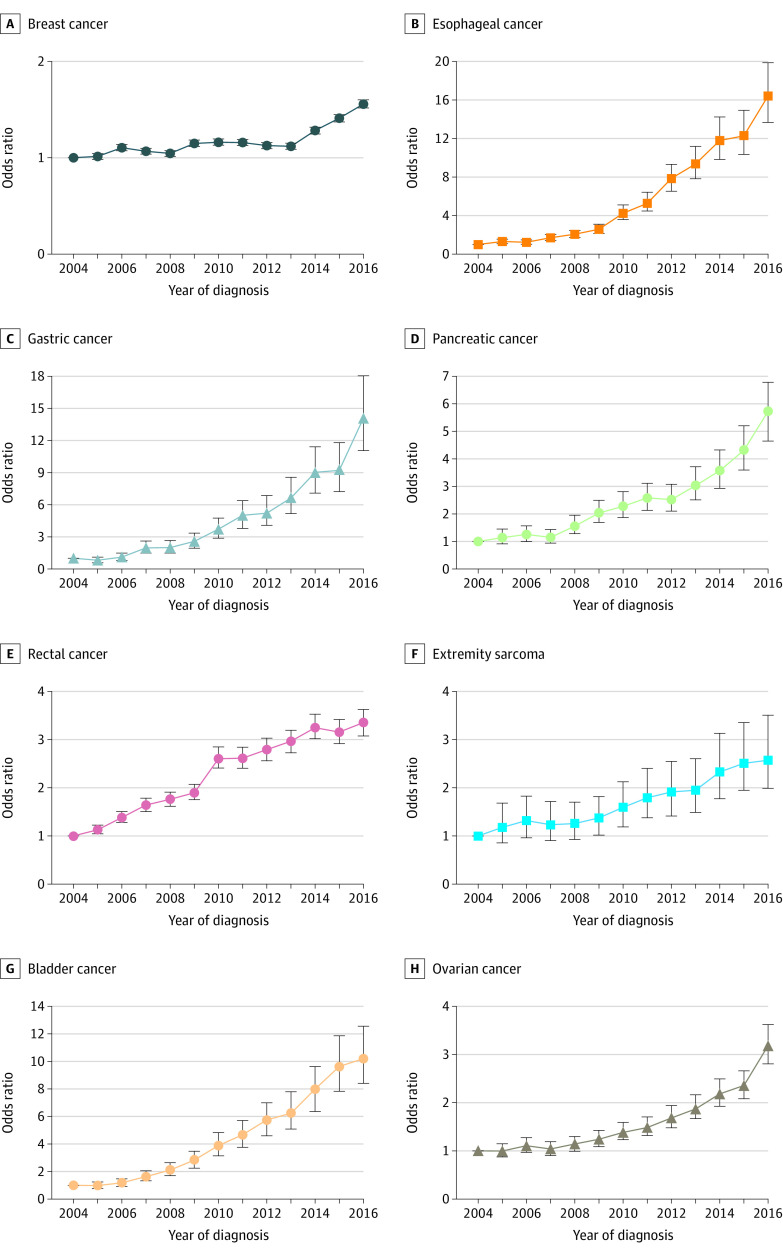
Trends in the Adjusted Odds of Receipt of Neoadjuvant Therapy by Cancer Type in the US From 2004 to 2016 Odds ratios for year of diagnosis were compared with the reference year (2004) after controlling for patient age, sex, race/ethnicity, educational level, income status, insurance type, distance from hospital, Charlson/Deyo Comorbidity Score, tumor grade, American Joint Committee on Cancer *Staging Manual, 7th Edition* pathologic TNM stage, procedure type, hospital annual organ-specific resection volume, hospital type, and hospital location. Error bars represent 95% CIs.

Of 2 292 734 cancer resections, 1 828 577 were for breast cancer, 46 210 for esophageal cancer 33 024 for stomach cancer, 61 514 for pancreatic cancer, 130 424 for rectal cancer, 32 908 for extremity sarcoma, 42 955 for bladder cancer, and 117 122 for ovarian cancer; 2 057 406 resections (89%) were performed in female patients. A significant increase in NT use was observed for each cancer type from 2004 to 2016 (Figure 1). After excluding patients with missing data (n = 188 609), 2 104 125 patients were included in the multivariable analyses. After controlling for patient, oncologic, and treatment hospital characteristics, the adjusted odds of NT increased over time for each cancer type with year of diagnosis entered as a categorical variable (Figure 2). Similar results were observed with year of diagnosis entered as a numeric variable. The odds ratio for breast cancer was 1.03 (95% CI, 1.02-1.04); esophageal cancer, 1.29 (95% CI, 1.27-1.30); stomach cancer, 1.26 (95% CI, 1.24-1.27); pancreatic cancer, 1.15 (95% CI, 1.14-1.17); rectal cancer, 1.11 (95% CI, 1.10-1.11); extremity sarcoma, 1.08 (95% CI, 1.07-1.10); bladder cancer, 1.24 (95% CI, 1.22-1.25); and ovarian cancer, 1.10 (95% CI, 1.09-1.11) (*P* < .001 for all).

## Discussion

Among centers accredited by the Commission on Cancer, NT use increased significantly for 8 different cancer types from 2004 to 2016, even after controlling for patient-, cancer-, and hospital-related factors. Although to our knowledge, this study is the largest to comprehensively evaluate NT trends in the US, the study was limited by inclusion of patients for whom NT may not have been indicated based on clinical staging and the inability to determine which patients initially had unresectable disease but whose tumors were downstaged after NT. Nevertheless, these observed trends have important implications. First, NT is multidisciplinary; thus, the need to overcome barriers to treatment delivery and to address disparities in NT use, especially for cancers in which NT is the standard of care, is urgent. Second, a better understanding of patient preferences, particularly among those who have cancers in which NT and upfront surgery have equipoise, is needed. Little is known regarding the degree of patient involvement concerning the sequence of surgery and systemic or radiation therapy.^6^ Third, little research has been conducted on patient quality of life during NT despite patients experiencing adverse effects of NT, adverse effects from the in situ cancer, and emotional effects related to “just getting the cancer out.” Future studies should focus on understanding the mechanisms behind these trends, improving the quality of evidence for each cancer type, and optimizing patient-centered research on NT.
